# Allele-specific disparity in breast cancer

**DOI:** 10.1186/1755-8794-4-85

**Published:** 2011-12-21

**Authors:** Fatemeh Kaveh, Hege Edvardsen, Anne-Lise Børresen-Dale, Vessela N Kristensen, Hiroko K Solvang

**Affiliations:** 1 Department of Genetics, Institute for Cancer Research, Norwegian Radium Hospital, Oslo University Hospital, Oslo, Norway; 2 Institute for Clinical Medicine, Faculty of Medicine, University of Oslo, Oslo, Norway; 3 Department of Clinical Molecular Biology (EpiGen), Medical division, Akerhus University Hospital, Lørenskog, Oslo, Norway; 4 Department of Biostatistics, Institute of Basic Medical Science, University of Oslo, Oslo, Norway

## Abstract

**Background:**

In a cancer cell the number of copies of a locus may vary due to amplification and deletion and these variations are denoted as copy number alterations (CNAs). We focus on the disparity of CNAs in tumour samples, which were compared to those in blood in order to identify the directional loss of heterozygosity.

**Methods:**

We propose a numerical algorithm and apply it to data from the Illumina 109K-SNP array on 112 samples from breast cancer patients. B-allele frequency (BAF) and log R ratio (LRR) of Illumina were used to estimate Euclidian distances. For each locus, we compared genotypes in blood and tumour for subset of samples being heterozygous in blood. We identified loci showing preferential disparity from heterozygous toward either the A/B-allele homozygous (allelic disparity). The chi-squared and Cochran-Armitage trend tests were used to examine whether there is an association between high levels of disparity in single nucleotide polymorphisms (SNPs) and molecular, clinical and tumour-related parameters. To identify pathways and network functions over-represented within the resulting gene sets, we used Ingenuity Pathway Analysis (IPA).

**Results:**

To identify loci with a high level of disparity, we selected SNPs 1) with a substantial degree of disparity and 2) with substantial frequency (at least 50% of the samples heterozygous for the respective locus). We report the overall difference in disparity in high-grade tumours compared to low-grade tumours (p-value < 0.001) and significant associations between disparity in multiple single loci and clinical parameters. The most significantly associated network functions within the genes represented in the loci of disparity were identified, including lipid metabolism, small-molecule biochemistry, and nervous system development and function. No evidence for over-representation of directional disparity in a list of stem cell genes was obtained, however genes appeared to be more often altered by deletion than by amplification.

**Conclusions:**

Our data suggest that directional loss and amplification exist in breast cancer. These are highly associated with grade, which may indicate that they are enforced with increasing number of cell divisions. Whether there is selective pressure for some loci to be preferentially amplified or deleted remains to be confirmed.

## Background

Higher organisms such as humans are diploid, which means that all chromosomes except the sex chromosomes are present in two copies (2 n). During DNA replication, unique genomic segments of original DNA are normally copied just one time, but in specific situations, these fragments are either not replicated entirely or they are copied more than once. The variation between normal and actual copy number of a DNA segment is referred to as copy number differences, which in normal cells are referred to as copy number variations (CNVs) and in tumour cells as copy number alternations (CNAs) [1]. Such chromosomal variations have been associated with genetic disorders (for CNVs) and the progression of cancer (for CNAs) [2]. The size of CNVs in normal tissues is often shorter than CNAs in tumour tissues, which can cover a large region of the human genome (Mb) [3]. Array-based comparative genomic hybridization (CGH) is used for detection of CNVs and CNAs [4,5].

Recently, new techniques have been developed to study CNVs and CNAs at a higher resolution using high-density arrays of single nucleotide polymorphisms (SNPs) [6]. SNPs are variations in the single-base locus of the genome within members of a species or paired chromosomes of an individual. In general, SNPs have precise positions on the chromosome and two alleles (maternal and paternal), and SNP-based arrays are powerful tools for detection of allele-specific chromosomal aberrations and loss of heterozygocity (LOH) status [7]. Recent advances in the systematic study of genomic alterations in human cancer samples have made it possible to understand this disease better and identify key genes with underlying roles in oncogenesis [8]. In the present study, we describe the development of a numerical algorithm based on Euclidian distances to extract the maximum level of information from SNP array data in blood-tumour pairs, with regard to both chromosomal aberrations and loss of heterozygosity. We apply the algorithm to Illumina 109K SNP array data and show how it can be used to mine information on a single-SNP, cytogenic region as well as in gene lists and at the pathway level. We focus on the disparity of CNAs in tumour samples, which were compared to those in blood in order to identify the directional loss of heterozygosity.

## Methods

### Materials

The 112 samples used here are a subset of a larger patient series consisting of 920 samples, which were collected from breast cancer patients to study the effect of disseminating tumour cells to the blood and bone marrow [9]. The samples were collected at five different hospitals in the Oslo region and have so far been extensively studied on both the clinical and biological levels [9,10].

### DNA isolation

Tumour tissues were snap-frozen at -80°C. Samples of mononucleus peripheral blood cells were drawn in tubes and centrifuged. Before freezing, the cell pellet was resuspended to a concentration of 30 × 10^6 ^cells/ml in a freezing solution containing RPMI added with 10% DMSO and 20% FCS as well as Dnase. The samples were then frozen at -70°C before being stored in liquid nitrogen. Before DNA isolation, 240 μl Prot.K (AB-Applied Biosystems) and 400 μl 1 × PBS without CaMg were added to the frozen samples on ice. The samples were incubated at 55°C until thawed and then transferred after mixing to the vessels in the Applied Biosystems 340A Nucleic Acid Extractor. The remaining process of DNA isolation followed the standard procedures for the extractor using a phenol-chloroform based method. DNA from frozen tumour tissue was extracted according to standard procedures of the Applied Biosystems 340A Nucleic Acid Extractor.

All patients provided informed consent, and the study was approved by the local regional ethical committees.

### Genotyping

The genotyping used has been previously described [11], and blood-tumour pairs were genotyped using the Human-1 109K BeadChip array (Illumina, San Diego, CA, USA). For each sample, the corresponding log R ratio (LRR) and B allele frequency (BAF) were extracted for both blood and tumour using the Illumina Beadstudio Genotyping software, which is based on the transformation of two channels' allelic intensity values [7]. Briefly Illumina's Beadstudio software performs normalization on the two raw allelic intensity values (*X, Y*) for each locus (SNP). *X *and *Y *values are respectively denoted as allele A and allele B. The normalized allele intensities are transformed to total signal intensity, *R *(*R *= *X *+ *Y*), and relative allelic signal intensity ratio, theta (θ = arctan(*Y*/*X*)(2/π)) [12].Illumina provides two supplementary parameters, LR*R *and *BAF*, which refer to the normalized and calibrated form of *R *and *θ*. BAF represents the proportion of one SNP allele (*B*) to the total number of *A *and *B *alleles (*N_B_*/(*N_A _*+ *N_B_*)) for each given loci. The Illumina Infinium platform detects chromosomal aberrations by comparing the normalized intensity of a subject sample and a reference sample using two modes of analysis: 1) a single-sample mode, which derives reference values from canonical genotyping clusters (0, 0.5, 1) created from clustering on normal reference samples; 2) Paired-sample mode, which makes intensity (R) comparisons between a subject sample and its paired reference sample. Genomic plots of LRR (LRR = log_2_(R_subject_/R_reference_)) and BAF of the three canonical clusters are used to detect chromosomal aberrations [1,13,14]. Comparing custom-clustered data to HapMap-clustered data, we observe improved scoring using custom clustering. The genotypes for both blood and tumour were therefore extracted using custom clustering [15]. To improve the results of genotyping for those samples that have not performed well, we used the custom-clustered data.

Missing values are denoted as NaN for both BAF and LRR. Updated SNP information (position and gene association) was derived when available using the UCSC Genome Browser (Human Mar. 2006 Assembly (NCBI36/hg18)) [16], dbSNP (build 130), Ensembl genome browser (Ensembl genes 57 database on Homo sapiens genes (GRCh37) dataset) [17], and CHIP Bioinformatics Tools, Goldenpath: hg18, dbSNP: build 130 [18].

### Description of computational algorithm

#### Pre-processing of data

Traditional Array CGH provides information on CNAs based on the *Log R *intensity ratios. The benefit of the Illumina SNP array analysis is that it provides additional information related to *B allele frequency*. The Beadstudio software presents information, based on two allelic intensity values, related to both *Log R ratio *and *B allele frequency*. To analyze the Illumina output, we propose a computational algorithm consisting of the following steps for each chromosome (illustrated by the flowchart in Additional file 1, Figure S1).

**Figure 1:**
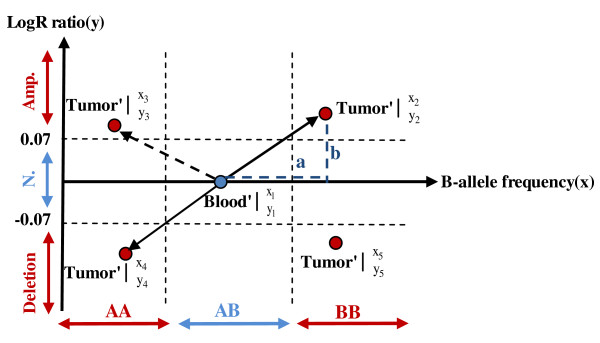
**Position of one blood sample in heterozygous**. The allelic disparity, amplification or deletion of one paired sample (blood-tumour) is illustrated in the heterozygous region. The position of one blood sample is denoted by (*x_1_,y_1_*) in the heterozygous region (AB). The same sample in the tumour can be either unaltered or shifted toward minor allele (AA-homozygous-amplification (*x_3_,y_3_*), AA-homozygous-deletion (*x_4_,y_4_*)), major allele (BB-homozygous-amplification (*x_2_,y_2_*) or BB-homozygous-deletion (*x_5_,y_5_*)). B-allele frequency is used for allelic disparity and Log R ratio for amplification or deletion.

We denote b_ij _and t_ij _as the blood sample and tumour sample, respectively. Here, i = 1,..., r and j = 1,..., c represent the number of loci and the number of samples, where r = 109352 and c = 112. Figure 1 illustrates the algorithm below for one locus. The Illumina data were pre-processed by sorting *BAF *and *LRR *values by chromosome and subsequently by position. All loci residing on chromosome Y were removed from our calculations. We denote *BAF *and *LRR *as × and y, respectively. We used the blood data as a reference and assumed a threshold to divide it into three regions (*AA*, *AB*, *and BB*). Monomorph loci and loci with less than three distinct genotype groups were considered non-informative and removed. The *BAF *value varies between 0 and 1 and the signal for the three genotypes on the *x *axis clusters around (*AA *~ 0, *AB *~ 0.5, *BB *~ 1); the assumed threshold covers samples in the following regions:

a. AA - homozygous: (0 ≤ b_ij _≤ 0.2)

b. AB - heterozygous: (0.2 < b_ij _≤ 0.8)

c. BB - homozygous: (0.8 < b_ij _≤ 1)

After filtering, we obtained 96,273 loci which modify index j into j = 1,..., r', where r' is the total number of loci without non-informative data.

### Allelic disparity

In the horizontal direction, for each locus of *Blood*', we obtained mean $\left(\bar{\mu_{B_{i}}}=\frac{1}{n}\overset{n}{\underset{j=1}{\sum }}b_{ij}\right)$ and standard deviation$(S_{B_{i}}=\sqrt{\frac{1}{n-1}\sum_{j=1}^{n}{\left(b_{ij}-\mu_{B_{i}}\right)}^{2})}.$ In the next step, heterozygous blood samples were extracted and labelled, as well as the entire tumour. To identify which tumour samples shifted from the heterozygous toward either one of the homozygous regions, we measured the distance (*D *= *x_B _- x_T_*) of associated samples in both the *x *and *y *axes, where *D *is the allelic disparity of *Blood*' and *Tumor*' samples. We deal only with those samples where $\mu_{B_{i}}-2S_{B_{i}}<D<\mu_{B_{i}}+2S_{B_{i}}$. For each locus, the frequencies of disparity of individuals were calculated. Here, let $n\left(t_{AA_{j}}\right)$ and $n\left(t_{BB_{j}}\right)$denote the numbers of samples for *j *shifted toward *AA*- and *BB*- homozygous, respectively. Based on the sample frequencies of each SNP, we defined a score (S) denoting the propensity of each SNP to lose either the B or A allele:

(1) $$S=\left\{\begin{array}{cc} 1-\frac{n\left(t_{BB_{j}}\right)}{n\left(t_{AA_{j}}\right)}.100 & \text{for}\;n\left(t_{AA_{j}}\right)>n\left(t_{BB_{j}}\right)\\ 1-\frac{n\left(t_{AA_{j}}\right)}{n\left(t_{BB_{j}}\right)}.100 & \text{for}\;n\left(t_{AA_{j}}\right)<n\left(t_{BB_{j}}\right) \end{array}\right)$$

We can apply and modify an arbitrary threshold via Score (*S*).

Furthermore, since we identify the number of samples shifted toward A or B-allele, we defined the percentage of samples shifted toward minor allele $\left(r\left(t_{BB_{j}}\right)\right)$, the percentage of samples shifted toward major allele $\left(r\left(t_{AA_{j}}\right)\right)$, and the percentage of heterozygous with disparity $\left(r\left(t_{AB_{j}}\right)\right)$, where

(2) $$r\left(t_{BB_{j}}\right)=\frac{n\left(t_{BB_{j}}\right)}{n\left(t_{AA_{j}}\right)+n\left(t_{BB_{j}}\right)}.100$$

(3) $$r\left(t_{AA_{j}}\right)=\frac{n\left(t_{AA_{j}}\right)}{n\left(t_{AA_{j}}\right)+n\left(t_{BB_{j}}\right)}.100$$

and

(4) $$r\left(t_{AB_{j}}\right)=\frac{n\left(t_{AA_{j}}\right)+n\left(t_{BB_{j}}\right)}{n\left(t_{AB_{j}}\right)}.100$$

The percentage of allele-specific disparity is given by

(5) $$\Delta_{j}=r\left(t_{BB_{j}}\right)-r\left(t_{AA_{j}}\right)$$

Based on the genotypes in blood, we tested for each SNP whether it fulfilled the criteria for *Hardy-Weinberg (HWE) *equilibrium [19]. Given one degree of freedom and a 0.95 confidence interval (CI), the threshold for the chi-square value is 3.841. Values equal to or below this threshold indicate that a locus is in HWE.

### Pathway and network analysis

Ingenuity Pathway Analysis (IPA) [20] version 9.0 (Release Date: 2011-03-22), Content version: 3206 (Release Date: 2011-02-11)) was used to analyse selected sets of genes in order to identify over-represented canonical pathways and networks. IPA core analysis allows us to find interactions between genes and proteins, related networks, functions and canonical pathways in the context of biological processes. The selected gene list was uploaded into IPA. The only filter used for the network analysis was "only consider molecules and/ or relationships where species = Human". The analysis included both direct and indirect relationships as well as endogenous chemicals, and for the network analysis the maximum number of molecules allowed per network was 140. In the network analysis, molecules of interest which interact with other molecules in the Ingenuity Knowledge Base are identified as Network Eligible molecules and serve as "seeds" for generating networks. The networks are scored with a numerical value. The score is based on 1) the number of Network Eligible molecules in the network, 2) size of the network, 3) Network Eligible molecules in the given dataset, and 4) the number of molecules in the IPA database that could potentially be included in the network. The network score is the -log(Fisher's Exact test result), which is based on the hypergeometric distribution. Network function displays the functional annotations associated with a specific network. Top networks represent associated network functions based on a score which reflects the negative logarithm of the p-value, which aggregates the likelihood of the genes in the network being found together due to random chance. Score ≥ 2 was considered significant. For the canonical pathways, the significance of the association between our defined data sets and the canonical pathways are assessed in two ways by the software: 1) the ratio of the number of molecules from our gene lists that map to a given canonical pathway and 2) False Discovery Rate (FDR) (*Benjamini *and *Hochberg *[21]) by the negative logarithm of B-H p-values indicating the likelihood that the association between the genes in our gene sets and a specific canonical pathway are explained by chance alone. Top biological functions were assigned to each network by the negative logarithm of B-H p-value, corrected for multiple testing (Source: IPA online manual).

### Chi-square test

The statistical chi-square test in R version 2.11.1 was used to estimate the association between the observed level of disparity and, among other, clinical and tumour-related parameters (TP53 mutation, ER, PR, HER2, Lymph node status, and subtypes of breast cancer).

### Cochran-Armitage trend test

The Cochran-Armitage trend test [21] is applied in R version 2.11.1 to detect the linear trend of ordered categorical data in a 2 × 3 contingency table. In this article, we used the Cochran-Armitage trend test for clinical parameters of grade and tumour size.

### Detection of amplification and deletion

**Figure 2:**
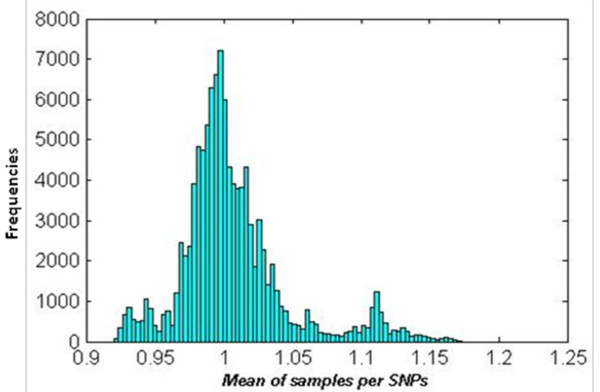
**Histogram of mean of samples per SNPs**. Mean of all samples per locus in a non-logarithmic PCF'ed tumour dataset to identify the proper threshold.

Based on the log R ratio, amplifications and deletions were detected with the following procedure: 1) LRR for tumour data were smoothed by the piecewise constant function (PCF) algorithm [22,23];2) Obtained PCF values were transformed into non-logarithmic values of smoothed LRR; 3) Mean of samples per locus was calculated for the data obtained in 2) and a histogram of the mean values was made (Figure 2); 4) To distinguish a proper threshold for amplification, deletion and normal region, three higher peaks were selected around 0.925, 1 and 1.12 in the non-logarithmic smoothed LRR; 5) Based on these three peaks, ±0.07 (logarithmic value of 0.95 and 1.05) was chosen as the threshold, where > 0.07 was classified as amplification and -0.07 ≤ normal ≤ 0.07 a nd < -0.07 as deletion (Figure 1).

## Results

In a two-dimensional space, a point is distinguished by two characters × and y; here, × is denoted by B allele frequency and y by Log R ratio (Figure 1). The two-dimensional position of one sample (*x_1_,y_1_*) in the heterozygous region (AB) in the blood (blue circle) is compared to its position in the tumour (red circle) to identify shifts toward AA-homozygous-Amplification (*x_3_,y_3_*), AA-homozygous-Deletion (*x_4_,y_4_*), BB-homozygous-Amplification (*x_2_,y_2_*), or BB-homozygous-Deletion (*x_5_,y_5_*). Horizontal shifting is shown by vector-a (B allele frequency) and vertical with vector-b (Log R ratio). By using the algorithm introduced in the Methods (Description of computational algorithm) on a dataset consisting of genotypes from 112 blood-tumour paired samples collected from breast cancer patients, we extracted information on changes in genotypes (allelic loss, here denoted as allelic disparity). The algorithm was applied separately to each autosomal chromosome as well as the × chromosome. In all, 96,273 loci were left after removing non-informative loci (see Methods on pre-processing of data) distributed across all chromosomes. The number of loci per chromosome varied between 1268 loci on chromosome 21 to 8559 on chromosome 1 (the numbers of SNPs and genes before and after filtering are given in Table 1 for each chromosome).

**Table 1:** Summary of studied loci and genes

	Original data			
	
Chromosome	Before Filtering		After Filtering	
	
	SNPs	gene	SNPs	gene
1	9819	1606	8541	1530
2	8702	1055	7718	1030
3	7207	891	6401	861
4	6000	649	5392	626
5	6329	738	5644	712
6	6579	858	5890	832
7	5581	730	4941	708
8	4891	559	4334	541
9	4480	601	3989	581
10	5240	614	4571	593
11	5928	1050	5310	1000
12	5465	881	4781	847
13	3093	288	2794	285
14	3420	501	3013	481
15	3307	477	2928	463
16	3388	624	2909	586
17	4079	896	3584	858
18	2570	236	2260	234
19	3520	1058	3157	1001
20	3007	477	2572	446
21	1381	218	1267	213
22	1886	358	1629	345
X	3480	598	2648	530

**Total**	**109352**	**15963**	**96273**	**15303**

Summary of all studied loci and genes in each chromosome before filtering of the data as well as the numbers of loci and genes showing disparity in both minor allele (AA) and major allele (BB) after removing the non-polymorphic or non-informative loci.

### Overall trends for allelic disparity in the genome

It is assumed that a locus has been subjected to allele-specific disparity if the signal from a heterozygous genotype shifts toward one or the other homozygous genotype at a distance larger than two standard deviations of the mean of the heterozygote for all samples. In order to identify the loci with the highest levels of disparity, we defined a score (S in eq. (1)) based on the sample frequency of asymmetrical shift for each heterozygous genotype, and all loci above or equal to 75 were selected (see Methods on allelic disparity), leaving 13,198 loci (Additional file 2, Figure S2). A full list of the 13,198 loci with information related to the genotypes in blood (number of samples in each genotype, number of samples in homozygote for both minor and major alleles, test for Hardy-Weinberg equilibrium, percent heterozygosity, number of missing genotypes, minor allele frequency, minor and major alleles) are given in Additional file 3, Table S1 as well as the information regarding genotypes in tumour (score (S), percentage and number of disparity toward minor or major allele (in eq. 2 and 3, respectively), percentage of heterozygote with disparity in eq. (4), percentage of allele-specific disparity in eq. (5) and preferential allele).

### Chromosomal regions enriched for allelic disparity

**Figure 3:**
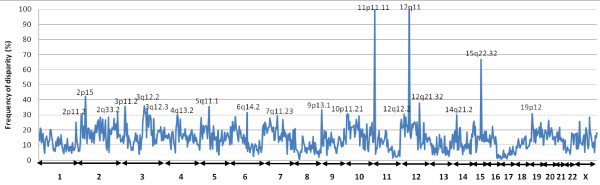
**Frequency of studied SNPs showing high degree of allelic disparity within a given cytoband**. Percentage of 13,198 loci according to cytoband localization.

In order to identify chromosomal regions with a high degree of disparity, we grouped the 13,198 loci according to cytoband localisation. The 13,198 loci fulfilling the criteria of score 75 or higher were distributed on 778 cytobands with 1-77 loci per cytoband. The cytobands having 30% and higher disparity are highlighted in Figure 3 (Table 2). Several loci have been previously reported in association with breast cancer.

**Table 2:** Cytoband SNP distribution of allelic disparity

Chr	Cytogenetic Band	Average of Score	Number of SNPs in cytoband	# of snps with allelic disparity (n = 13198)	Represented genes with disparity
11	11p11.11	100,0	1	1	OR4C46
12	12q11	85,7	1	1	CPNE8
15	15q22.32	77,5	3	2	SMAD6
2	2p15	91,2	85	36	CCT4,COMMD1,KIAA1841,LOC51057,MDH1,TMEM17,UGP2,USP34,XPO1
12	12q21.32	90,8	29	11	Cep290,FLJ35821,HGNT-IV-H,KITLG
3	3q12.2	90,4	50	18	ABI3BP,GPR128,IMPG2,NIT2,TFG,TOMM70A
3	3p11.1	89,5	42	15	CGGBP1,EPHA3,HTR1F,MGC26717,ZNF654
5	5q11.1	86,6	31	11	EMB,PARP8
3	3q13.13	89,6	108	37	CD96,DPPA2,DPPA4,DZIP3,ESRRBL1,GUCA1C,HHLA2,KIAA1524, MORC,PVRL3,TCRIM
9	9p13.1	83,3	3	1	FLJ35740
3	3q13.11	90,6	72	23	ALCAM,CBLB,LOC131368
2	2q33.2	84,8	38	12	ABI2,ALS2CR13,ALS2CR14,ALS2CR16,CD28,CYP20A1,ICOS,RAPH1
6	6q14.2	85,7	19	6	SNAP91,PGM3,KIAA1117,PRSS35,NCB5OR
3	3q12.3	92,2	67	21	FAM55C,IMPG2,LOC131368,NFKBIZ,PCNP,RPL24,SENP7
10	10p11.22	91,6	97	30	ARHGAP12,C10orf68,CCDC7,EPC1,ITGB1,KIF5B,NRP1,PARD3,TCF8
19	19p12	88,1	81	25	LOC148213,LOC163223,LOC91120,ZNF100,ZNF15L1,ZNF208,ZNF254, ZNF429,ZNF43,ZNF430, ZNF91
3	3p11.2	88,2	13	4	DKFZP564O123,HTR1F
12	12p12.2	89,4	36	11	PDE3A,SLCO1B1,SLCO1B3,SLCO1C1
2	2p12	88,2	181	55	C2orf3,CTNNA2,FLJ13391,HK2,LRRTM1,LRRTM4,MRPL19,PAP,POLE4, REG-III,SUCLG1,TACR1
4	4q13.2	87,5	100	30	BRDG1,CENPC1,DESC1,DKFZp686L1818,EPHA5,FLJ10808,FLJ16046, FLJ21934,GNRHR,HAT,LOC339967,UGT2B10,UGT2B4,UGT2B7,YT521
14	14q21.2	93,8	70	21	BTBD5,C14orf106,C14orf155,FKBP3,KIAA0423,LRFN5,PRPF39,RPL10L

Chromosomal regions with a high degree of disparity per cytoband (S ≥ 7 5)

### Single-SNP allelic disparity

**Figure 4:**
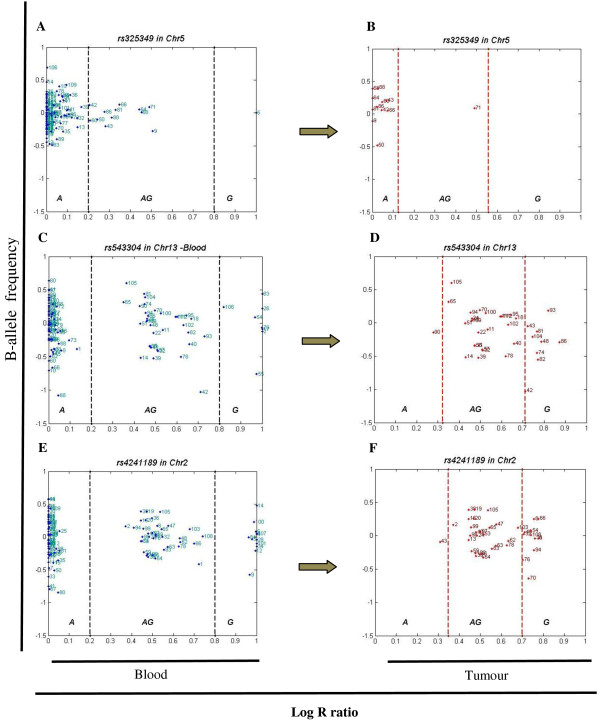
**Three SNPs with high levels of allelic disparity**. Panel (A) illustrates the B allele frequency for *rs325349 *in blood for all samples (A = 99, AG = 12 and G = 1) on chromosome 5 located in *CDH9*. Out of the 12 samples scored as AG in the blood, Panel (B). Black dash lines show the arbitrary thresholds for AA (major allele, A), AB (AG) and BB (minor allele, G) regions, and red dash lines are the real thresholds of heterozygous. Only one sample is left; 91.7 percent (n = 11) of heterozygous samples show disparity in tumour and all shifted toward major allele (A). Panel (C) shows the same information for SNP, *rs543304 *on chromosome 13 located in *BRCA2 *for blood (A = 70, AG = 34, and G = 8), and panel (D) shows this information for tumour; 81.8 percent of heterozygous samples (n = 8) shifted toward minor allele (G). Panel (E) shows B-allele frequency for rs4241189 on chromosome 2 in *HECW2 *gene, and panel (F) shows that for tumour.

To further identify loci with a high degree of disparity, we looked at the single-SNP level (Additional file 3, Table S1). For all SNPs, we counted the number of alleles in our dataset and obtained the actual genotype. A compelling example is the SNP rs325349 (A/G) on chromosome 5 located in *CDH9*, for which 91.7 percent (n = 11) of the heterozygous samples showed disparity in the tumour and all shift in one direction, toward the major allele (A) (Figure 4 A and 4B). SNP rs543304 located in *BRCA2 *on chromosome 13 also showed a high level of disparity, where 30 percent of the studied samples were heterozygous with 26 percent displaying disparity (n = 9), the majority toward the minor allele (G, n = 8) (Figure 4 C and 4D). Another example is rs4241189 located in *HECW2 *on chromosome 2, where 33 percent of the samples were heterozygous, 28 percent (n = 11) showing disparity (primarily toward the minor allele (G), n = 10) (Figure 4 E and 4F).

### Allelic disparity in relation to clinical parameters

**Figure 5:**
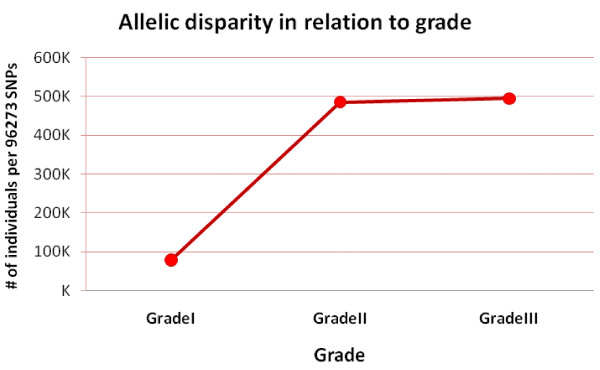
**Allelic disparity in relation to clinical parameters**. Trend of disparity in each individual locus related to the clinical parameter of grade.

We observed significantly different frequencies of allelic disparity events according to grade, from well differentiated (G1) to poorly differentiated (G3) breast cancers (p-value < 0.001 (Table 3)). Histologic grade is based on the microscopic appearance of the cancer cells, which are graded into four categories: Grades 1, 2, 3 and 4. Grade 1 tumours are well-differentiated and resemble normal cells; these tumours tend to grow slowly and are considered less aggressive. Grade 2 tumours are moderately differentiated, but grade 3 and 4 tumours tend to differentiate poorly and grow rapidly [24]. Figure 5 shows the trend from well differentiated (G1) toward poorly differentiated (G3) for the disparity level of samples.

**Table 3:** Clinical features

Grade			
**Status**	**Grade I**	**Grade II**	**Grade III**

Disparity (96273 loci)	79120 (5.9%)	485506 (9.3%)	494383 (12.6%)

No Disparity (96273 loci)	1268281 (94.1%)	4711144 (90.7%)	3451422 (87.4%)

Total	1347401 (100%)	5196650 (100%)	3945805 (100%)

**p-value < 0.001**, (Cochran-Armitage trend test = X-squared = 68885.98, df = 1)			

**ER**			

**Status**	**ER = neg**	**ER = pos**	

Disparity (96273 loci)	471696 (11.7%)	611716 (9.3%)	

No Disparity(96273 loci)	3570454 (88.3%)	5932211 (90.7%)	

Total	4042150 (100%)	6543927 (100%)	

**p-value < 0.001**, (Pearson's Chi-squared test, X-squared = 14659.47, df = 1)			

**HER2**			

**Status**	**HER2 = neg**	**HER2 = pos**	

Disparity (96273 loci)	820891 (10.4%)	191389 (9.9%)	

No Disparity(96273 loci)	7070289 (89.6%)	1733669 (90.1%)	

Total	7891180 (100%)	1925058 (100%)	

**p-value < 0.001**, (Pearson's Chi-squared test, X-squared = 14659.47, df = 1)			

**TP53**			

**Status**	**TP53 = wildtype**	**TP53 = mutate**	

Disparity (96273 loci)	607219 (8.2%)	483065 (14.3%)	

No Disparity(96273 loci)	6802862 (91.8%)	2885412 (85.7%)	

total	7410081 (100%)	3368477 (100%)	

**p-value < 0.001**, (Pearson's Chi-squared test, X-squared = 96216.44, df = 1)			

**PR**			

**Status**	**PR = neg**	**PR = pos**	

Disparity (96273 loci)	659139 (12%)	409458 (8.2%)	

No Disparity(96273 loci)	4826429 (88%)	4594835 (91.8%)	

Total	5485568 (100%)	5004293 (100%)	

**p-value < 0.001**, (Pearson's Chi-squared test, X-squared = 42038.96, df = 1)			

Distribution of the samples according to the clinical features such as grade, tumour size, ER, PR, HER2 status, breast cancer subtypes, and TP53 mutation status between the SNPs showing disparity and those not showing disparity.

Allelic disparity was also found to be associated with HER2, ER, PR and TP53 mutation status. A significant higher level of disparity was found in samples with negative status for either one of the receptors HER2, ER, or PR (Table 3). Samples harbouring mutations in TP53 were also more likely to have a high degree of disparity compared to samples with wild type TP53 (Table 3).

To focus on specific genes in considering the allelic disparity of individual loci, we used only the loci in which the event was observed in 50 percent or more of the samples, leaving 176 loci (in 158 unique genes, indicated in Additional file 3, Table S1). Additional file 4, Table S2 shows loci with high levels of disparity associated with the clinical or molecular parameters such as stage, grade, Her2 and TP53 mutation status. We applied multiple testing corrections. Since the number of loci was 176 (less than 1000), we summarized p-values in Additional file 4, Table S2. Several genes have shown significant association in clinical features: *AGA*, *BANP*, *BCOR*, *CACNB1*, *CSMD1*, *CUBN*, *CXCR4*, *DERL2*, *DNMT3B*, *EDNRB*, *EPB41L3*, *FAM20A*, *FLJ31882*, *FLJ33008*, *FLJ43339*, *FLJ45831*, *GEMIN4*, *GGTLA1*, *GHITM*, *HNT*, *HTR3B*, *IGSF4*, *KIAA1043*, *KLF15*, *KREMEN1*, *LOC51255*, *LOC81691*, *MGC21654*, *MYCBP2*, *NDST4*, *NGL-1*, *NVL*, *PIK3R5*, *PPP1R9B*, *RTN4IP1*, *SLC2A9, SLC43A2, SPAG6, ST7, STK32B, TRPS1, TXNL1 *and *ZW10*. Tumour size is classified into four sub-categories: T1 (Tumour is 2 cm or less across), T2 (Tumour is more than 2 cm but not more than 5 cm across), T3 (Tumour is more than 5 cm across) and T4 (Tumour of any size growing into the chest wall or skin) [25]; here, T3 and T4 are merged in one category. We found allelic disparity in these chromosomal loci: 2p11.2, 4p16.2, 4q26, 7q31.2, 10p12.2, 10p13, 11q23.2, 11q25, 13q22.3, 15q26.3 and 17p13.3. We also analyzed the two subtypes of breast cancer, luminal epithelial-like subtype A and basal epithelial-like subtype [26] and we found evidence for allelic disparity in the following chromosomal loci: 8q24.13, 13q22.3, 17p12, 17p13.2, 1q42.12, 17q25.1, 22q11.23, 15q15.1 and 16p12.3. The corresponding genes are given in Additional file 4, Table S2.

### Allelic disparity and pathway analysis

**Figure 6:**
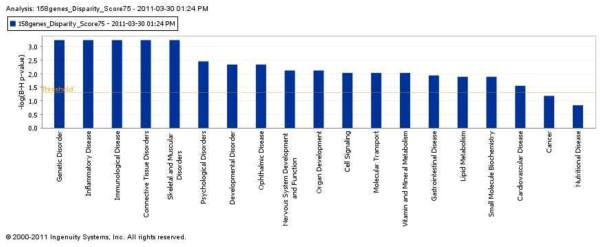
**Bio-functions analysis identified 17 bio-functions as significantly enriched within the genes with a high level of disparity (Score ≥75)**. Top bio functions in Ingenuity Pathway Analysis (IPA).

In the list of 176 loci that show a high level of allelic disparity, we used IPA to perform a core analysis of the gene set and identify the most significantly associated canonical pathways (CP) as well as over-represented networks (Table 4). The top bio functions in IPA are shown in Figure 6. Table 4 illustrates the top three network functions, related bio functions, IPA-score, p-values, and number of molecules for 1) lipid metabolism, small molecule biochemistry, etc. (IPA-score 158); 2) genetic disorder, lipid metabolism, etc. (IPA-score = 52); 3) cell death, cellular development, etc. (IPA-score = 24).

**Table 4:** Top associated network functions and bio functions

Associated Network Functions	Score	
**Lipid Metabolism**, Small Molecule Biochemistry, Nervous System Development and Function	158	
Genetic Disorder, **Lipid Metabolism**, Ophthalmic Disease	52	
Cell Death, Cellular Development, Renal Regeneration	24	

**Disease and Disorders**	**P-Value**	**# Molecules**

Genetic Disorder	3.35E-07 - 4.97E-02	96
Inflammatory Disease	8.40E-07 - 4.59E-02	57
Immunological Disease	1.47E-06 - 4.16E-02	51
Connective Tissue Disorders	1.57E-06 - 4.16E-02	40
Skeletal and Muscular Disorders	1.57E-06 - 4.59E-02	55

**Molecular and Cellular Functions**	**P-Value**	**# Molecules**

Cell Signaling	9.30E-05 - 4.35E-02	17
Molecular Transport	9.30E-05 - 4.91E-02	24
Vitamin and Mineral Metabolism	9.30E-05 - 4.32E-02	15
Lipid Metabolism	1.59E-04 - 4.97E-02	8
Small Molecule Biochemistry	1.59E-04 - 4.97E-02	19

Top three associated network functions and related scores from IPA. Top bio functions contain 1) diseases and disorders, 2) molecular and cellular functions.

### Allelic disparity and stem cell genes

An overview of the gene sets used in this study is given in Additional file 5, Figure S3. For each gene set, we extracted the reported genes and compiled then into one stem cell gene list. After removing overlapping redundant information, this list contained 2899 genes. We then applied a standard chi-square test to compare the observed level of allelic disparity between the stem cell gene list and the list of non-stem cell genes (n = 13,198), as well as the levels of amplification and deletion. Genes were scored as disparity genes if more than 50% of loci related to a given gene showed disparity (score ≥75) (genes w here t he SNPs were e qual ly distributed were not included, Table 5A). We did not see a significantly higher level of disparity overall in loci associated with the stem cell genes compared to the non-stem cell genes when "collapsed" by associated or closest gene (p-value = 0.8008). We also analysed overall copy number changes between the two gene-sets (stem cell and non-stem cell) and observed that the stem cell genes are more often altered by deletion than by amplification compared to the non-stem cell genes (p-value = 5.41E-04, Table 5B).

**Table 5:** Chi-square test results for stem cell

A. allelic Disparity - Score 75			
**Gene level**			
	
	**Non-Stem Cell genes**	**Stem Cell genes**	**P-value**

**No Disparity**	11315 (95%)	2514 (95%)	0.8008
**Disparity**	615 (5%)	140 (5%)	
**Total**	11930 (100%)	2654 (100%)	

**B. Amplification and Deletion**			

**Gene level**			
	
	**Non-Stem Cell genes**	**Stem Cell genes**	**P-value**

**Amplification**	5594 (51%)	1159 (47%)	0.000541
**Deletion**	5432 (49%)	1313 (53%)	
**Total**	11026 (100%)	2472 (100%)	

A 2x2 table showing observed results from the allelic disparity (A) and amplification or deletion (B) gene lists.

## Discussion

### Chromosomal regions enriched for allelic disparity

We first identified chromosomal regions which were enriched for allelic disparity. Several of these loci have been previously reported in association with breast cancer. For instance, the genetic variation in 5q11.1 has previously been associated with the risk of breast cancer in multiple GWAS studies [27]. Bergamschi *et al*. reported 5q11.1 to be more frequently deleted in basal-like subtype tumours [28]. In the same study, they also found 3q12.2 loss to be associated with Luminal B tumours [28,11]. Chromosomal aberrations of 3q13.13 have been detected in several types of cancer, including breast, gastric, colon, and squamous cell carcinomas of head and neck; furthermore, *KIAA1524 *residing in this region has been associated with an aggressive form of breast cancer [29].

### Single-SNP allelic disparity

Next, we further identified the SNPs that most strongly contributed to allelic disparity. Such SNPs were, for instance, SNP rs543304 located in *BRCA2*, rs325349 located in *CDH9*, and rs4241189 located in *HECW2*. Abnormalities or mutations of *BRCA2 *gene have been linked to increased risk of both familial breast and ovarian cancer [29]. The encoded protein is involved in repairing cell damage and maintaining normal growth of breast cells [29]. *CDH9 *encodes a type II classical cadherin from the cadherin family. The cadherins are integral membrane proteins important for cell-cell adhesion [30]. *HECW2 *encodes an E3 ubiquitin-protein ligase and mediates ubiquitination of TP73, a p53 family member regulating cell growth and apoptosis. The enzyme acts to stabilize TP73 and enhance activation of transcription by TP73 [31].

### Allelic disparity in relation to clinical parameters

Allelic disparity was observed in association to grade (degree of differentiation), HER2, ER, PR and TP53 mutation status in a number of genes.

SNP rs1041242 found associated with stage is located in the *MYCBP2 *gene on chromosome 13 (q22.3). MYCBP2 is known as a probable E3 ubiquitin-protein ligase which mediates ubiquitination and subsequent proteasomal degradation of target proteins. MYCBP2 may function as a facilitator or regulator of transcriptional activation by *MYC *[29]. Three SNPs in the gene *ST7 *on the 7q31.1-q31.2 region were also found associated with stage. *ST7 *has been reported as a tumour-suppressor gene involved in a variety of other human cancers and cell lines derived from breast tumours have been shown to harbor mutations in *ST7 *[32]. Locus rs3824271 found associated with ER status is located in the *CSMD1 *gene on chromosome 8p23.2, a region reported to be deleted in up to 50% of breast cancers [33]. Deletion of 8p23.2 has been associated with high histologic grade [34].

### Biological pathway analysis (IPA)

The main network functions identified through pathway analysis were lipid metabolism, small molecule biochemistry, genetic disorder, lipid metabolism, cell death, and cellular development. Activation of lipid metabolism has been reported to be an early event in carcinogenesis [35] and many studies at the single-gene level of lipid metabolism have revealed an effect on tumour genesis [36].

### Stem cells

One of the most important characteristics of an embryonic stem cell is its ability of self-regeneration and differentiation. If one assumes that the initial targets of deregulation will be among the stem cell genes, then one may expect that they will represent the "oldest" events in an unstable genome, which would be reinforced in every cell division. We therefore hypothesized that the stem cell genes were likely to have a higher level of disparity in the tumour [37,38]. To test this, we extracted the stem cell genes from 13 published gene sets [39-41]. This hypothesis, however, was not confirmed, suggesting that the observed allelic disparity is perhaps not merely a function of the number of divisions after a single event but the result of a more complex and subtle evolutionary process.

## Conclusion

Breast cancer is one the most frequent malignant and a multifactorial disease affecting women, and thus it is reasonable to hypothesize that there is an association between multiple genomic rearrangements and tumour sample disparities. Since most tumours diverge from the diploid level, many studies have reported only gains and losses by array CGH.

Our data suggest that directional loss and amplification exist in breast cancer. These are highly associated with grade, which may indicate that they are enforced with increasing number of cell divisions. Whether there is a selective pressure for some loci to be preferentially amplified or deleted remains to be confirmed.

## Competing interests

The authors declare that they have no competing interests.

## Authors' contributions

FK designed computational algorithm and programming. VNK and HKS designed the study. ALBD and VNK financed and conducted data acquisition. VNK, HE and HKS were involved in data analysis. FK, HE, VNK and HKS wrote the manuscript. All authors have read and approved the final manuscript.

## Pre-publication history

The pre-publication history for this paper can be accessed here:

http://www.biomedcentral.com/1755-8794/4/85/prepub

## Supplementary Material

Additional file 1 **Figure S1 - Flowchart of methods**. Two outputs of Illumina BeadStudio, B allele frequency (BAF on × axis) and Log R ratio (LRR on y axis) were used as input. A threshold was applied to BAF of blood to cover the three canonical AA, AB and BB genotypes. If the number of samples in each region was equal to 112, then SNP was removed as non-informative data. Both informative blood and tumour data were retrieved as Blood' & Tumour'. Mean standard deviation and variances of Blood' heterozygous were obtained. Then movement of Tumour' samples was compared to that of the Blood' heterozygous samples, and if the measured distance was equal to or greater than µBi ± 2σBi, the frequencies of samples moved toward AA/BB regions were measured. A flexible score (S) was calculated and the symmetrical regions were eliminated. Involved genes (84881 SNPS) of the asymmetric region were extracted. The Hardy-Weinberg equilibrium (HWE), the chi-square test with one degree of freedom, and 5% significance levels of values were calculated. Those SNPs in the HWE region were flagged. For the horizontal disparity, among 84,881 SNPs we selected SNPs with a score of 75 or more (n = 13198). From this list we selected SNPs if 50% or more of the samples of heterozygote blood shifted (n = 176 SNPs, representing 158 genes). Click here for file

Additional file 2 **Figure S2 - Disparity of SNPs with score ≥ 75 in chromosomes 1-23**. Disparity of loci with scores greater than or equal to 75. Blue colour shows SNPs that shifted toward minor allele (AA), and red colour shows SNPs that shifted toward major allele (BB). Click here for file

Additional file 3 **Table S1 - Loci with highest level of allelic disparity**. This supplementary table contains 13,198 loci that show allelic disparity. The column of "SNP information" includes loci, gene, chromosome, cytogenetic band, stem cells and top 176 loci distinguished by IPA. The columns for "Blood" show the numbers of major allele (BB), heterozygous, minor allele (AA), percentage of heterozygous, total number of samples, minor allele frequency (MAF), and results of HWE chi-square test. The column of "Tumour" contains percentage of heterozygous with disparity obtained by eq. (4), percent of allele-specific disparity by eq. (5), preferential allele, score, number of samples shifted toward major allele and its percentage by eq. (3), and number of samples shifted toward minor allele and its percentage by eq.(2). Click here for file

Additional file 4 **Table S2 - Significant loci related to the clinical features with highest level of allelic disparity**. Allelic disparity of individual loci in relation to clinical parameters such as tumour size, ER, PR, TP53 mutation, HER2 status, Basal-Lum A subtypes, grade, and lymph node status was analyzed. Click here for file

Additional file 5 **Figure S3 - Overview of stem cell gene sets**. Thirteen gene sets of stem cells are compiled in one list and compared for allelic disparity and amplification or deletion. Click here for file
