# Supplementary figures and images for: Allele-specific disparity in breast cancer
Source: BMC Med Genomics. 2011 Dec 21;4:85. doi: 10.1186/1755-8794-4-85 (PMC3337547; doi:10.1186/1755-8794-4-85)

# Additional file 1, Figure S1 – Flowchart of methods

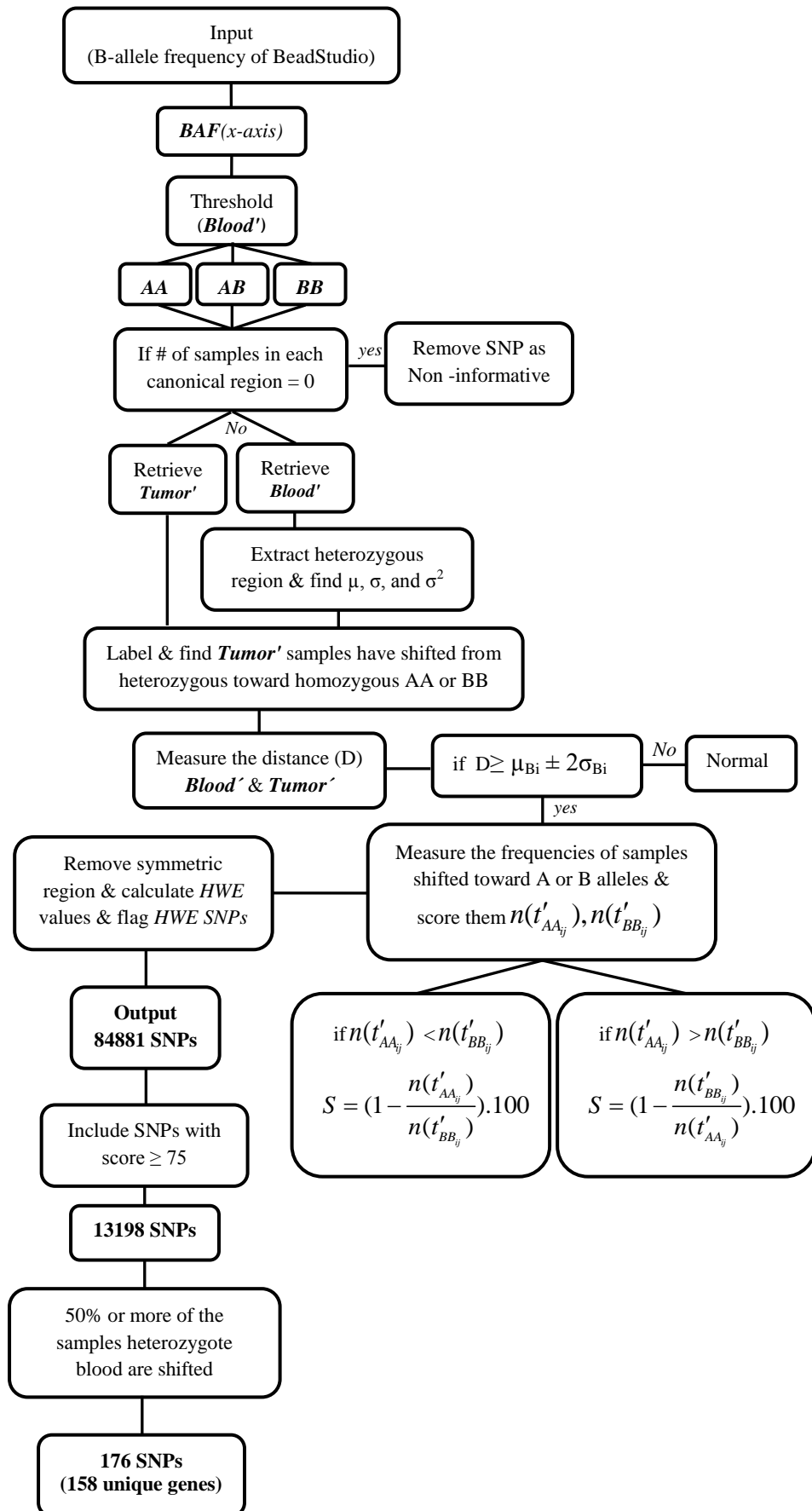

Supplement: Additional file 1 — Figure S1 - Flowchart of methods. Two outputs of Illumina BeadStudio, B allele frequency (BAF on × axis) and Log R ratio (LRR on y axis) were used as input. A threshold was applied to BAF of blood to cover the three canonical AA, AB and BB genotypes. If the number of samples in each region was equal to 112, then SNP was removed as non-informative data. Both informative blood and tumour data were retrieved as Blood' & Tumour'. Mean standard deviation and variances of Blood' heterozygous were obtained. Then movement of Tumour' samples was compared to that of the Blood' heterozygous samples, and if the measured distance was equal to or greater than µBi ± 2σBi, the frequencies of samples moved toward AA/BB regions were measured. A flexible score (S) was calculated and the symmetrical regions were eliminated. Involved genes (84881 SNPS) of the asymmetric region were extracted. The Hardy-Weinberg equilibrium (HWE), the chi-square test with one degree of freedom, and 5% significance levels of values were calculated. Those SNPs in the HWE region were flagged. For the horizontal disparity, among 84,881 SNPs we selected SNPs with a score of 75 or more (n = 13198). From this list we selected SNPs if 50% or more of the samples of heterozygote blood shifted (n = 176 SNPs, representing 158 genes). [file 1755-8794-4-85-S1.PDF]

**Additional file 2, Figure S2 – Disparity of SNPs with score  $\geq 75$  in chromosomes 1-23**

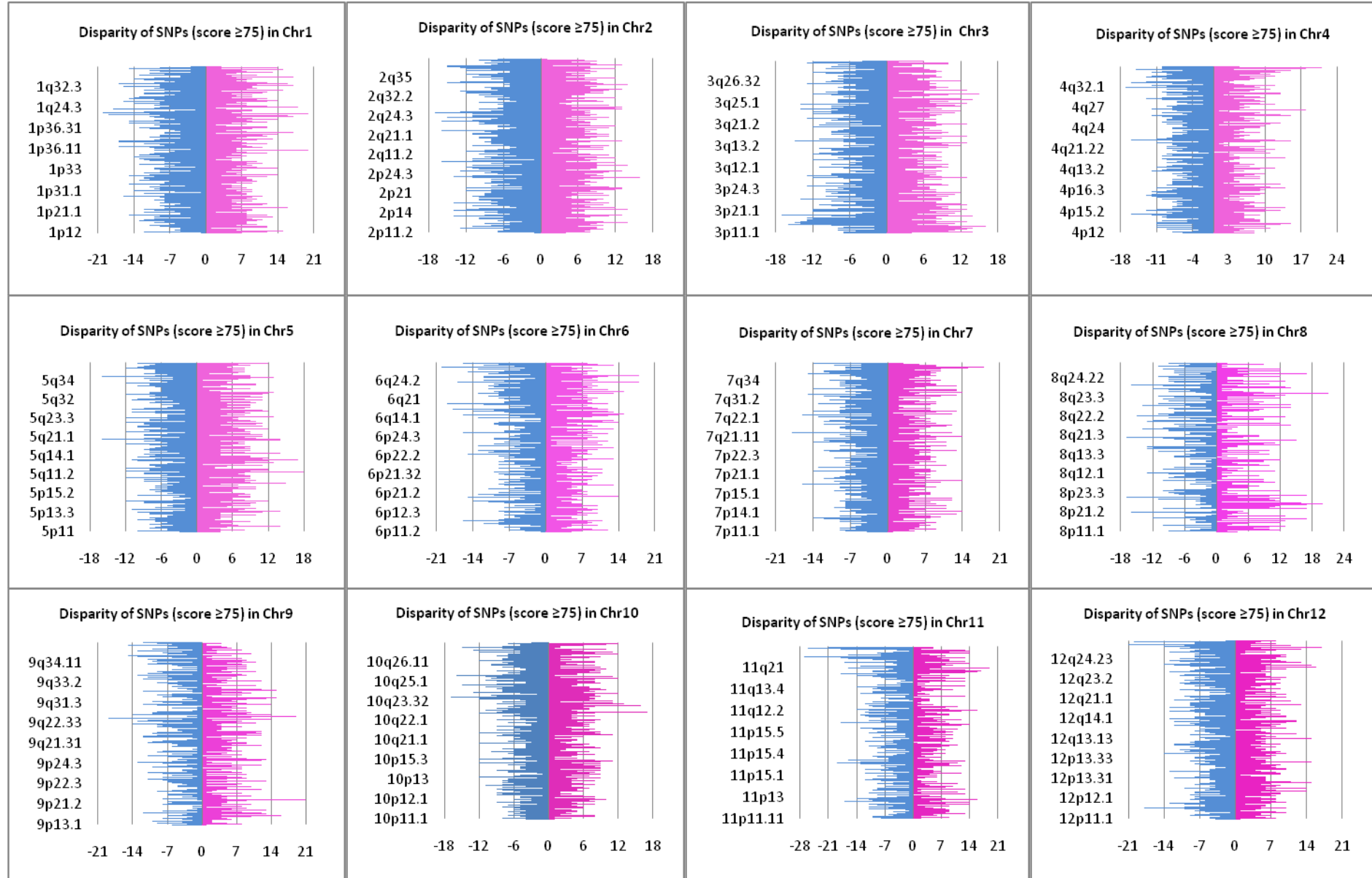

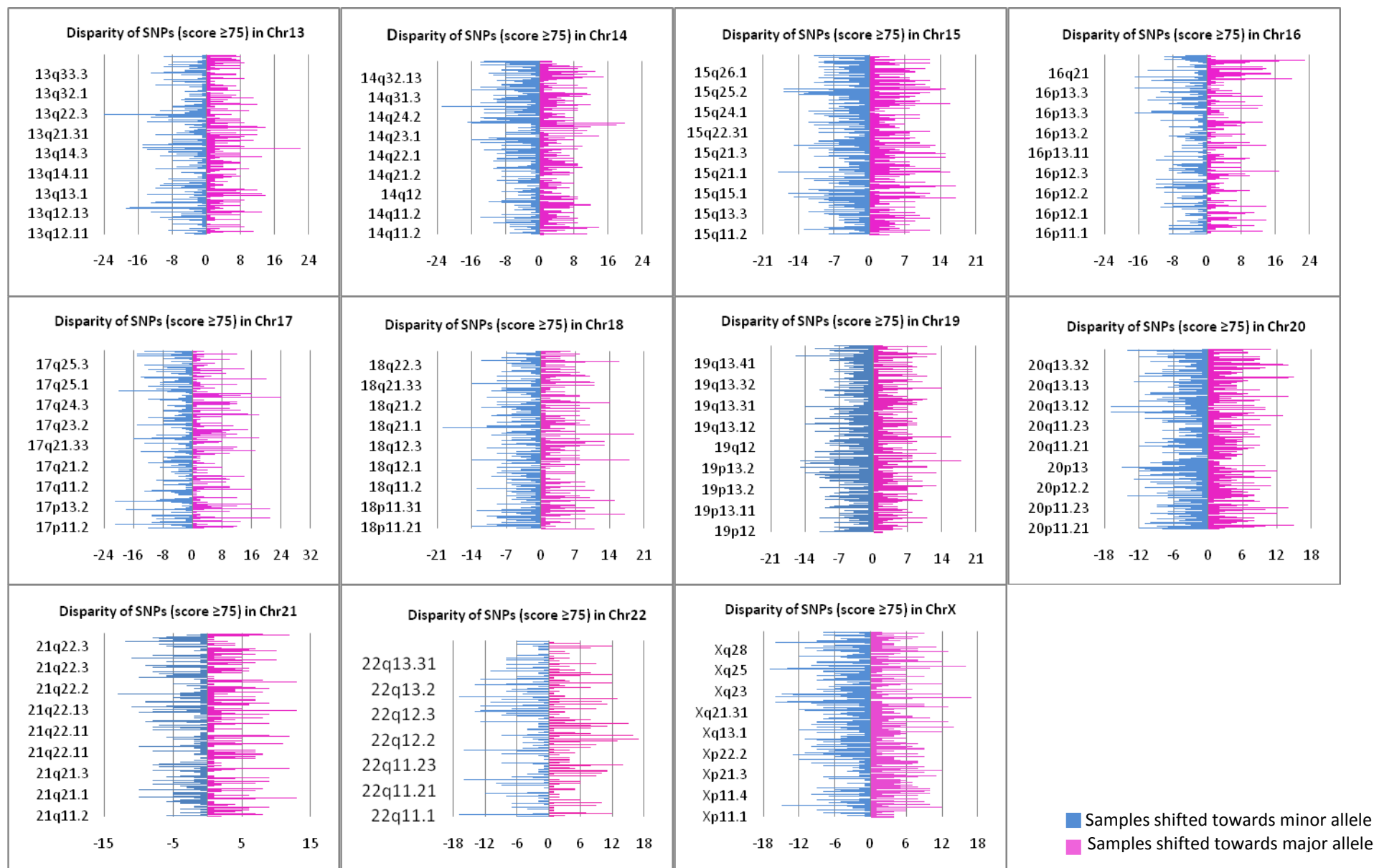

Supplement: Additional file 2 — Figure S2 - Disparity of SNPs with score ≥ 75 in chromosomes 1-23. Disparity of loci with scores greater than or equal to 75. Blue colour shows SNPs that shifted toward minor allele (AA), and red colour shows SNPs that shifted toward major allele (BB). [file 1755-8794-4-85-S2.PDF]

Additional file 5, Figure S3 – Overview of stem cell gene sets

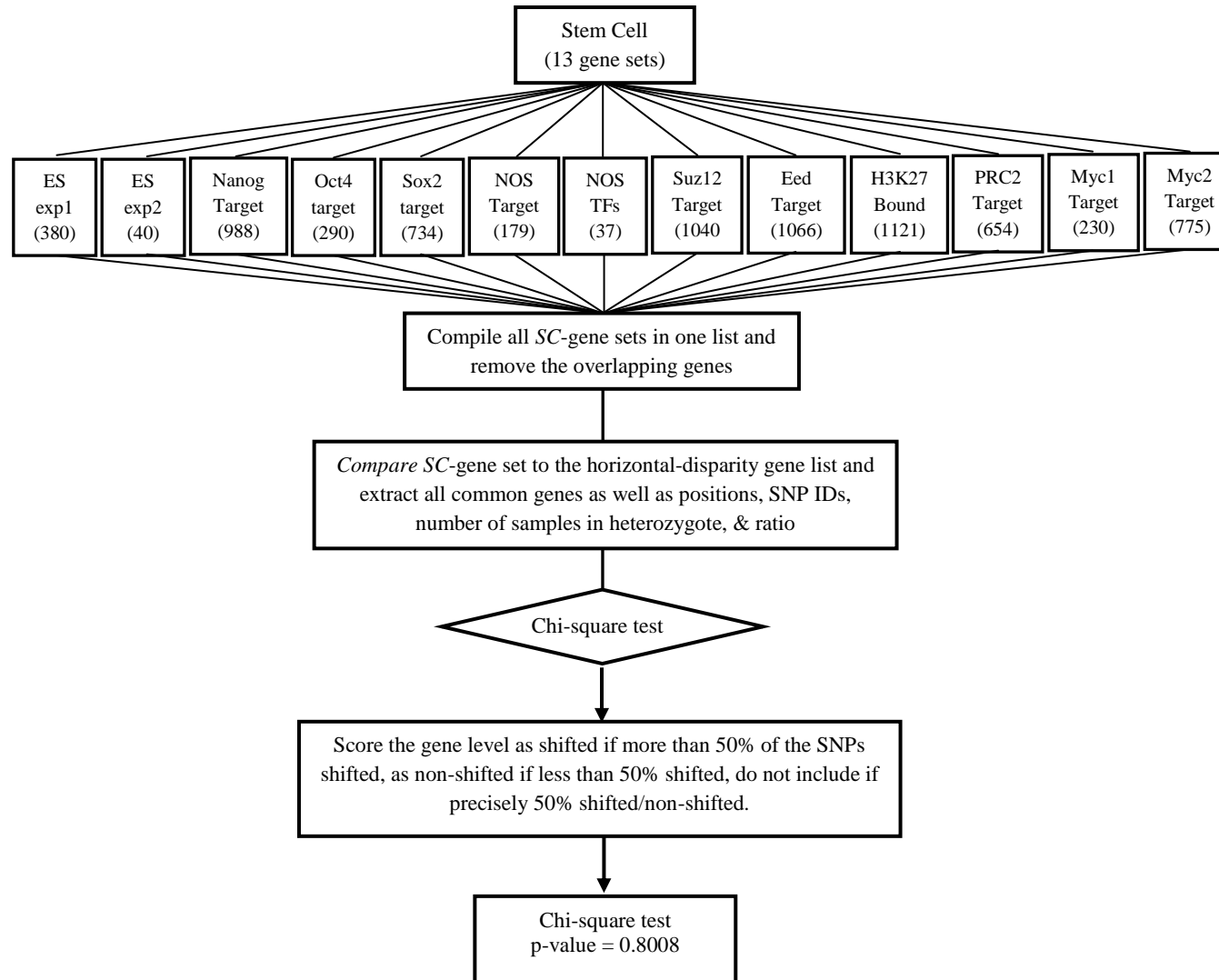

Supplement: Additional file 5 — Figure S3 - Overview of stem cell gene sets. Thirteen gene sets of stem cells are compiled in one list and compared for allelic disparity and amplification or deletion. [file 1755-8794-4-85-S5.PDF]
